# Persistent hyperglycemia is an independent predictor of outcome in acute myocardial infarction

**DOI:** 10.1186/1475-2840-6-2

**Published:** 2007-02-06

**Authors:** Iwan CC van der Horst, Maarten WN Nijsten, Mathijs Vogelzang, Felix Zijlstra

**Affiliations:** 1 Department of Cardiology, University of Groningen Medical Center, University of Groningen, The Netherlands; 2 Intensive Care Medicine, University of Groningen Medical Center, University of Groningen, The Netherlands

## Abstract

**Background:**

Elevated blood glucose values are a prognostic factor in myocardial infarction (MI) patients. The unfavourable relation between hyperglycemia and outcome is known for admission glucose and fasting glucose after admission. These predictors are single measurements and thus not indicative of overall hyperglycemia. Increased persistent hyperglycemia may better predict adverse events in MI patients.

**Methods:**

In a prospective study of MI patients treated with primary percutaneous coronary intervention (PCI) frequent blood glucose measurements were obtained to investigate the relation between glucose and the occurrence of major adverse cardiac events (MACE) at 30 days follow-up. MACE was defined as death, recurrent infarction, repeat primary coronary intervention, and left ventricular ejection fraction equal to or smaller than 30%.

**Results:**

MACE occurred in 89 (21.3%) out 417 patients. In 17 patients (4.1%) it was a fatal event. A mean of 7.4 glucose determinations were available per patient. Mean +/- SD admission glucose was 10.1 +/- 3.7 mmol/L in patients with a MACE versus 9.1 +/- 2.7 mmol/L in event-free patients (P = 0.0024). Mean glucose during the first two days after admission was 9.0 +/- 2.8 mmol/L in patients with MACE compared to 8.1 +/- 2.0 mmol/L in event free patients (P < 0.0001). The area under the receiver operator characteristic curve was 0.64 for persistent hyperglycemia and 0.59 for admission glucose. Persistent hyperglycemia emerged as a significant independent predictor (P < 0.001).

**Conclusion:**

Persistent hyperglycemia in MI has a stronger relation with 30-day MACE than elevated glucose at admission.

## Background

Acute myocardial infarction (MI) patients with hyperglycaemia at admission have a worse prognosis than patients that are normoglycemic at admission [1,2]. This relation is found in both patients with diabetes mellitus (DM) and in patients without DM [3-6]. It has been argued that in patients without DM hyperglycaemia may be caused by undetected diabetes [7,8]. Some patients who present with hyperglycaemia are indeed diabetic but many patients with an admission glucose above 11.0 mmol/L are not diabetic [9]. Elevated fasting glucose after admission for MI also predicts an unfavourable outcome [10-12]. Admission glucose and fasting glucose as predictors of outcome have the drawback that they are based on a single measurement and thus are not indicative of persistent hyperglycaemia [1-5,10,11,13,14]. A first step to define persistent hyperglycaemia is to compute the mean of all glucose values. However, an ordinary arithmetic mean of glucoses ignores the unequal time distribution between measurements [14]. The calculation of the time-averaged glucose addresses this problem. In this post-hoc analysis of data collected in a prospective study of MI patients we sought to investigate whether persistent hyperglycemia, defined as an elevated time-averaged glucose over the first 48 hours after admission is a better predictor of major adverse cardiac events than elevated glucose at admission.

## Subjects and methods

### Subjects

All patients with symptoms consistent with an acute MI of >30 min duration, presenting within 24 hour after the onset of symptoms and with a ST-segment elevation of more than 1 mm (0.1 mV) in two or more contiguous leads on the electrocardiogram and treated with primary percutaneous coronary intervention (PCI) were included in this study. At baseline age, gender, previous cardiovascular disease defined as a history of coronary artery bypass grafting (CABG), previous PCI, stroke and MI, existence of DM, smoking status, Killip class, electrocardiographic site of infarction, time of onset of symptoms, and time of hospital admission were recorded. Patients were defined as diabetic when treated with a diet, oral hypoglycaemic drugs and/or insulin. The research protocol was reviewed and approved by the medical ethics committee, and patients were included after informed consent.

### Glucose measures

Glucose levels were based on measurements of whole-blood glucose (Modular System, Roche/Hitachi, Basel, Switzerland). The first glucose available after admission was defined as admission glucose. To determine the time-averaged glucose level for an individual patient, a dedicated computer algorithm interpolated all glucose measurements into a curve, after which the area under this glucose curve was calculated for the first 48 hours (persistent hyperglycemia). The area under the curve was then divided by 48 hours.

### Enzymatic infarct size

Enzymatic infarct size was estimated by serial measurements of creatine kinase (CK) fraction. CK was determined enzymatically on a Hitachi 717 automatic analyzer according to the International Federation of Clinical Chemistry (IFCC) recommendation at 30 degrees Celsius. Frequent CK determinations were performed according to a schedule that called for 4 to 8 measurements in the first 96 hours to calculate the area under the CK curves.

### Left ventricular function

Left ventricular ejection fraction (LVEF) was measured before discharge by radionuclide ventriculography or by echocardiography. Radionuclide ventriculography was performed with the multiple-gated equilibrium method following the labeling of red blood cells of the patient with technetium (^99m^Tc-pertechnate). A General Electric 300 gamma-camera with a low-energy all-purpose parallel-hole collimator was used. Global ejection fraction was calculated by a Star View computer (General Electric, Wisconsin, USA) using the fully automatic PAGE program. LVEF as assessed by two-dimensional transthoracicechocardiography was reported as a descriptive grade of function, using subjective visual assessment by two independent observers. This approach is less time-consuming than other methods, such as Simpson's rule or the wall motion index score. Nevertheless, studies of subjective visual assessment of LVEF suggest that this approach can be at least as accurate as other methods [15]. A LVEF ≤ 30% was defined as a poor left ventricular function before the analysis [16].

### Cardiac events

In all patients data were obtained with respect to mortality, recurrent myocardial infarction or repeat PCI during the first 30 days after admission. The primary endpoint of the study was the presence of a major adverse cardiac event (MACE) during the first 30 days after admission for acute MI. MACE was defined as the composite incidence of death, recurrent infarction, repeat PCI or a LVEF ≤ 30%. The combination of death and non-fatal major events as recurrent infarction, repeat intervention, and heart failure has been shown to be a valid predictor of 1-year mortality [17,18]. If in one patient more than one event occurred, these events accounted for one MACE. Recurrent myocardial infarction was defined as the occurrence of symptoms consistent with an acute MI of >30 min duration, signs of infarction on the electrocardiogram, and a second increase in serum CK level to more than twice the upper limit of normal. If the CK level had not decreased to normal levels, a second increase of more than 200 IU per litre over the previous value was regarded as indicative of a recurrent infarction [19]. Repeat PCI was defined as angioplasty performed within 30 days due to repeat signs and symptoms of myocardial ischemia. If an event occurred within 48 hours after admission persistent hyperglycemia was calculated based on the values available before the event.

### Statistical analysis

Differences between groups were assessed with the Student's *t*-test or the Mann-Whitney *U*-test. The Chi-square test and the Chi-square test for trend were used to test differences between proportions. Receiver operator characteristic (ROC) curves were computed to assess the ability of glucose-derived parameters to predict MACE or mortality. We also performed a Cox proportional-hazards regression model with factors at admission related to MACE at least with a single-sided level of significance of 0.2 or less. The Statistical Package for the Social Sciences (SPSS Inc., Chicago, IL, USA) version 11.5 was used for all statistical analysis.

## Results

Between April 1, 1998 and October 1, 2001, 417 patients were included. After 30 days a MACE had occurred in 89 patients (21.3%); in 17 patients (4.1%) it was a fatal event and in 72 patients (17.3%) a non-fatal event. A repeat PCI was performed in 20 patients (4.8%). Left ventricular function data were available of 377 patients (90.4%). In only 6 patients (1.4%) a reinfarction occurred within 30 days, whereas in 68 patients (18%) the LVEF was equal or smaller than 30% was observed. Baseline demographic and clinical characteristics of patients with a MACE and patients without an event are represented in table 1 and table 2. A mean of 7.4 glucose determinations were available per patient. Mean ± SD admission glucose was 10.1 ± 3.7 mmol/L in patients with a MACE versus 9.1 ± 2.7 mmol/L in event-free patients (P = 0.0024) (table 3). Mean persistent hyperglycemia was 9.0 ± 2.8 mmol/L in patients with MACE compared to 8.1 ± 2.0 mmol/L in event-free patients (P < 0.0001). A total of 44 patients had DM, of whom in two cases DM had not been diagnosed before. In A glucose level at admission in the highest quartile was observed in 34 DM patients (77.3%) and 38 DM patients (86.4%) had persistent hyperglycemia.

**Table 1:** Baseline characteristics patients with a major adverse cardiac events (MACE) and without an adverse event within 30-days

Characteristics	MACE group	Event-free group	P-value
Number of patients	89	328	
Age, years (mean ± SD)	62.5 ± 12.6	60.1 ± 11.8	0.11
Men	70 (78.7)	263 (80.2)	0.77
Previous cardiovascular events	17 (19.1)	47 (14.3)	0.17
Diabetes mellitus	11 (12.4)	33 (10.1)	0.33
Type 2 diabetes mellitus	11 (12.4)	28 (8.5)	0.29
Tablets	6 (6.7)	17 (5.1)	0.82
Insulin	2 (2.2)	13 (3.4)	
Hypertension	32 (36.0)	87 (26.5)	0.06
Dyslipidaemia	16 (18.0)	75 (22.9)	0.20
Currently smoker	41 (46.1)	178 (54.3)	0.11
Positive family history	27 (30.3)	133 (40.5)	0.05

Data are numbers (%) unless otherwise indicated.

**Table 2:** Infarct characteristics, hemodynamic status and reperfusion treatment

Characteristics	MACE group	Event-free group	P-value
Number of patients	89	328	
Systolic blood pressure, mmHg (mean ± SD)	125 ± 23	132 ± 21	0.03
Diastolic blood pressure, mmHg (mean ± SD)	76 ± 17	78 ± 14	0.13
Heart rate (mean ± SD)	79 ± 15	73 ± 16	0.02
Anterior MI	69 (77.5)	131 (39.9)	<0.001
Killip class 1	79 (88.8)	311 (95.0)	0.04
Killip class ≥ 2	10 (11.2)	17 (5.0)	
Multi-vessel disease	58 (65.2)	156 (47.6)	0.002
TIMI grade 0 flow before PCI	60 (67.4)	212 (64.6)	0.36
Stent	48 (53.9)	184 (56.1)	0.82
GP IIb/IIIa receptor blocker	28 (26.2)	79 (24.1)	0.33
TIMI grade 3 flow after PCI	75 (86.2)	305 (93.3)	0.03

Data are number (%) unless otherwise indicated.

**Table 3:** Relation between glucose measures and major adverse cardiac events

	MACE group	Event-free group	P-value
Number of patients	89	328	
Admission glucose	10.1 ± 3.7	9.1 ± 2.7	0.0024
Persistent hyperglycemia	9.1 ± 2.8	8.0 ± 2.0	<0.0001

Glucose data expressed as mmol/L (mean ± SD).

The area under the ROC-curve for admission glucose was 0.59 (95% confidence interval 0.52–0.65) and for persistent hyperglycemia was 0.64 (0.57–0.70). Factors related with MACE in univariate analysis were previous cardiovascular events, hypertension, dyslipidemia, smoking, positive family history, anterior site of infarction, Killip class ≥ 2, multi-vessel disease, TIMI grade 3 flow after PCI, admission glucose, and persistent hyperglycemia (table 1 and 2; all P <= 0.2). In the Cox proportional hazard model, after correction for these factors anterior site of infarction (hazard ratio (95% confidence interval) 4.06 (2.46–6.68), P < 0.001), Killip class ≥ 2 (1.97 (1.02–3.83), P = 0.045), multi-vessel disease (1.99 (1.28–3.09), P = 0.002), TIMI grade 3 flow after PCI (2.33 (1.26–4.31), P = 0.007) and persistent hyperglycemia (1.12 (1.04–1.20), P = 0.003) remained independent prognostic factors of MACE. In the subgroup of patients without known diabetes mellitus also an independent relation of persistent hyperglycemia with 30-day MACE could be observed (1.19 (1.05–1.36), P = 0.006).

**Figure 1:**
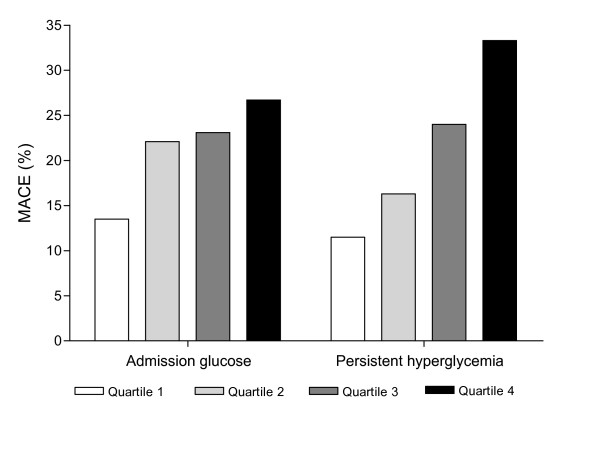
30-day major adverse cardiac events (MACE) according to quartiles of admission glucose and persistent hyperglycemia in MI patients. P value for trend in admission glucose is 0.023 and for persistent hyperglycemia is <0.0001.

The positive relation between admission glucose and MACE is illustrated in figure 1. In the lowest quartile of admission glucose MACE occurred in 13.5% compared to 26.7% in the highest quartile (P for trend 0.023). That persistent hyperglycemia has a stronger relation with 30-day MACE than admission hyperglycemia is also illustrated in figure 1. The stepwise increase is more pronounced with persistent hyperglycemia than with admission hyperglycemia. The lowest quartile of persistent hyperglycemia was 11.5% compared to 33.3% in the highest quartile (P for trend < 0.0001).

30-day mortality increased with each quartile of persistent hyperglycemia, with 1 patient that had died in the lowest quartile versus 9 patients (8.6%) in the highest quartile (table 4). All patients in the lowest quartile for admission glucose survived and 8 patients (7.7%) and 7 patients (6.7%) in the two highest quartiles did not survive the first 30 days after admission (table 5).

**Table 4:** Relation of quartiles of persistent hyperglycemia with major adverse cardiac events, mortality and infarct size

	Quartile 1	Quartile 2	Quartile 3	Quartile 4	P-value*
Glucose range (mmol/L)	5.4–6.9	7.0–7.6	7.7–8.8	8.9–20.6	
MACE (%)	12 (11.5)	17 (16.3)	25 (24.0)	35 (33.3)	<0.0001
30-day mortality	1 (1.0)	2 (1.9)	5 (4.8)	9 (8.6)	0.003
LVEF (% ± SD)	45.3 ± 9.8	43.3 ± 9.8	40.9 ± 12.3	39.4 ± 13.2	<0.001
CK AUC (IU ± SD)	919 ± 669	1430 ± 1547	1560 ± 1307	1579 ± 1402	<0.001

MACE = major adverse cardiac events. LVEF = left ventricular ejection fraction. SD = standard deviation. CK = creatine kinase. AUC = area under the curve. *P value denotes P value for trend.

**Table 5:** Relation of quartiles of admission glucose with major adverse cardiac events, mortality and infarct size

	Quartile 1	Quartile 2	Quartile 3	Quartile 4	P-value*
Glucose range (mmol/L)	4.3–7.2	7.3–8.5	8.6–10.3	10.4–22.9	
MACE (%)	14 (13.5)	23 (22.1)	24 (23.1)	28 (26.7)	0.023
30-day mortality (%)	0 (-)	2 (1.9)	8 (7.7)	7 (6.7)	0.003
LVEF (% ± SD)	44.6 ± 10.6	41.9 ± 11.4	41.8 ± 11.7	40.7 ± 12.0	<0.001
CK AUC (IU ± SD)	1001 ± 771	1283 ± 975	1505 ± 1349	1690 ± 1783	<0.001

MACE = major adverse cardiac events. LVEF = left ventricular ejection fraction. SD = standard deviation. CK = creatine kinase. AUC = area under the curve. *P value denotes P value for trend.

The relation between admission glucose and persistent hyperglycemia and reduced LVEF and increased enzymatic infarct size is strong (table 4 and 5). In the quartiles of admission glucose the LVEF was 44.6%, 41.9%, 41.8% and 40.7%, and in persistent hyperglycemia the LVEF was 45.3%, 43.3%, 40.9% and 39.4% (both P < 0.0001). The mean ± SD enzymatic infarct size was 1001 ± 771 IU in the lowest quartile of admission glucose versus 1690 ± 1783 IU in the highest quartile (P < 0.0001) and 919 ± 669 IU in the lowest quartile of persistent hyperglycemia compared to 1579 ± 1402 IU in the highest quartile (P < 0.0001).

## Discussion

In this study we observed that persistent hyperglycemia in acute MI has a stronger relation with unfavourable short-term outcome than glucose at admission. Previous studies have primarily focused on the prognostic value of admission hyperglycaemia in both patients with and without DM [1,2,5,13,20]. Some studies showed that an elevated fasting glucose after admission also predicts unfavourable outcome [10-12]. Only one study used a measure of persistent hyperglycaemia in the analysis of the relation between deregulation of the glucose metabolism during MI [14]. In a study of 662 MI patients hyperglycaemia was defined as the presence of a glucose level on admission or a 4-day mean blood glucose level higher than 6.67 mmol/L. A total of 457 patients (69.0%) had hyperglycaemia and only 195 (29.7%) had previously known diabetes mellitus. These patients developed more complications, and had higher 28-day mortality [14]. The relation of persistent hyperglycemia was observed both in the overall population as in the subgroup of patients without known diabetes.

### Mechanisms of action

Several mechanisms can be postulated to explain the relation between acute and persistent hyperglycaemia and an unfavourable outcome after reperfusion therapy for MI. First, the relation between persistent hyperglycaemia and outcome may be related to the presence of ongoing stress in more severely ill patients. During myocardial ischemia an increase in glucose levels is observed [21]. It has been reported that glucose was related to adrenalin and cortisol in patients with ST-segment elevation MI [22]. In the current study, Killip class ≥ 2 on admission was more frequent and enzymatic infarct size was larger, suggesting that hyperglycaemia reflects extensive myocardial damage.

The second possible explanation is that patients with hyperglycaemia are likely to have diabetes, even if it has not been diagnosed [7-9,23]. It has been described that patients with DM have an impaired outcome after MI. Potentially, patients with persistent hyperglycaemia are more likely to have DM either known or unknown [9,11]. In our study, patients with DM had indeed an elevated glucose at admission and a persistent hyperglycemia. The number of patients without DM in the highest quartile of admission glucose and persistent hyperglycemia was approximately 2 out of 3 patients. Diabetes is associated with extensive coronary artery disease and adverse outcomes in MI patients. In the current study, elevated admission glucose and persistent hyperglycemia were related to a higher prevalence of DM. Persistent hyperglycemia may reflect insulin resistance. Insulin resistance is more often present in non-diabetic patients with an acute MI compared to matched controls [24]. When 181 patients admitted with acute MI and no history of diabetes mellitus were compared to 180 matched controls without previously known diabetes or cardiovascular disease, glucose, HbA1c, proinsulin, proinsulin/insulin ratio, triglycerides, insulin resistance and fibrinogen were all consistently higher in patients than controls (P < 0.01). Recently, in a retrospective study, it was shown that non-diabetic patients with signs of insulin resistance had impaired left ventricular function recovery after coronary angioplasty compared to patients without insulin resistance [25].

Finally, it is possible that hyperglycemia and concomitant metabolic abnormalities may exacerbate myocardial damage in MI. A recent study in 4102 non-diabetic patients admitted for heart failure found a strong association between admission glucose and short-term mortality [26]. As in our study, the heart failure study does not allow conclusions whether hyperglycemia was an indicator or cause of adverse outcome.

### Role for insulin infusion

The obvious clinical difference between admission glucose and persistent hyperglycemia is that hyperglycemia during hospital stay is amenable to therapy, whereas hyperglycemia already present on admission cannot be modified. Therefore, in the clinical setting, the ultimate proof that the hyperglycemic state may have harmful effects on the ischemic myocardium is to demonstrate that cardiac outcomes can be improved by strict glucose regulation. The beneficial effect of an infusion of insulin during myocardial ischemia has been suggested by a small number of clinical studies, although they lacked a randomized design and were not conclusive [27-29]. The only large trials to investigate treatment focussed on hyperglycemia in MI patients were the Diabetes Insulin-Glucose in Acute Myocardial Infarction (DIGAMI) studies. The DIGAMI included 620 patients with known diabetes mellitus or serum-glucose concentrations of over 11.0 mmol/L [30,31]. Patients were randomized for an insulin-glucose infusion for 24 hours, followed by a minimum of 3 months of intensive insulin therapy or conventional treatment. After 1 year, patients that had no previous insulin treatment gained most by glucose-metabolism intervention, with a mortality of 8.6 versus 18.0% in the control group. For the study population as a whole, the authors found an absolute reduction in mortality of 7.5%. The recently published DIGAMI 2 randomized 1253 MI patients with hyperglycemia to 24 hour insulin-glucose infusion to obtain glucose levels between 7.0 and 10.0 mmol/L and subcutaneous long-term insulin treatment thereafter, to 24 hours glucose control followed by standard glucose control thereafter, or to standard glucose control started after admission [32]. After a follow-up of 2 years no difference in mortality could be observed: 23.4% versus 21.2% and 17.9% (NS). Glucose levels at admission averaged 12.7 mmol/L and fall to 9.1 mmol/L in the intensive treatment group compared to 10.0 mmol/L after standard treatment. The drop in glucose level of 3.4 mmol/L obtained in DIGAMI 2 was thereby smaller than in their first trial (5.8 mmol/L). Moreover, in the smaller Hyperglycemia: Intensive Insulin Infusion In Infarction (HI-5) study with 240 patients, the mean blood glucose level after 24 hours was 8.3 mmol/L in the treatment group compared to 9.0 mmol/L in the conventional group (NS) [33]. A mean insulin dose administered over this time in the treatment group was (only) 1.9 units/hours. An effect on mortality could not be observed.

The relation between hyperglycemia and outcome could be one of the explanations for the lack of benefit of trials using insulin in combination with glucose-potassium, i.e. GIK [29,34-37]. Patients randomized to GIK suffered more often from hyperglycemia after 24 hours. Future trials have to determine whether or not to aim for strict glucose regulation during acute MI, since in critically ill patients it has been studied and proven to be effective in critically ill patients [38,39]. The level of glucose levels obtained (4.4–6.1 mmol/L) were much lower than in both DIGAMI, HI-5 and GIK trials.

### Study limitations

Although in this analysis persistent hyperglycemia depicted 30-day MACE over admission glucose the area under the ROC curves for both measures were small. Other factors than glucose deregulation must account for unfavourable outcome after MI. On the other hand admission glucose is seen as one among other measures that help to predict outcome and possibly persistent hyperglycemia could serve as a better prognostic value. The number of patients in our analysis is not large, although the level of significance for observed relations was strong. The number of patients with diabetes was too small to draw conclusions on the relation of persistent hyperglycemia and outcome in the subgroup of patients with diabetes mellitus. It would have been useful if we had been informed about the degree of chronic hyperglycemia before the myocardial infarction, as reflected by glycated hemoglobin (HbA_1c_). Unfortunately, we did not systematically record HbA_1c_. Diabetes was diagnosed after admission in two patients, although the true number of diabetics might still have been higher. Finally, in a recent study, fasting glucose was superior to admission glucose with regard to 30-day mortality [12]. We did collect glucose measures according to a time-scheme but had no information whether the measures collected during the morning were in the fasting state. Therefore, we could not perform an analysis on the relation of fasting glucose.

## Conclusion

In patients with acute MI treated with primary PCI, hyperglycemia defined by the time-averaged glucose during the first 48 hours of hospital stay predicts unfavourable short-term outcome better than admission hyperglycemia. Persistent hyperglycemia could serve as a tool to compare algorithms to obtain more strict glucose control in MI patients, as well as in other critically ill patients. Possibly, it could serve as a tool to recognize patients with unknown diabetes. It is of great interest to see whether studies directed to lower glucose levels after admission lead to a reduction of unfavourable events and preservation of infarct-size.

## Competing interests

The author(s) declare that they have no competing interests.

## Financial disclosures

None declared.
